# Real-World Outcomes of Ipilimumab–Nivolumab vs. Anti-PD-1 Monotherapy in Metastatic Uveal Melanoma: A Single-Center Retrospective Study

**DOI:** 10.3390/cancers17213521

**Published:** 2025-10-31

**Authors:** Gitta Pánczél, Patrik Horváth, Erijona Temaj, Kata Czirbesz, Mihály Tamás Kispál, Georgina Fröhlich, Tímea Balatoni

**Affiliations:** 1 Department of Dermatology, National Institute of Oncology, 1122 Budapest, Hungary; horvath.patrik@oncol.hu (P.H.); temaj.erijona@oncol.hu (E.T.); czirbesz.kata@oncol.hu (K.C.); kispal.mihaly@oncol.hu (M.T.K.); balatoni.timea@oncol.hu (T.B.); 2 Centre of Radiotherapy, National Institute of Oncology, 1122 Budapest, Hungary

**Keywords:** uveal melanoma, metastatic uveal melanoma, immune checkpoint inhibitors, ipilimumab, nivolumab, real-world evidence, progression-free survival, immune-related adverse events

## Abstract

**Simple Summary:**

Uveal melanoma is a rare eye cancer with a high metastatic potential, especially to the liver. Until very recently, no effective systemic therapy was available, and prognosis for patients with metastatic disease remained dismal. Anti-PD-1 monotherapy has shown minimal activity in this setting, whereas the combination of ipilimumab plus nivolumab can induce higher tumor response and disease control rates, although at the cost of substantially greater toxicity. Our study reports real-world outcomes from a single cancer center, illustrating both the potential and the limitations of current immunotherapeutic strategies for metastatic uveal melanoma.

**Abstract:**

Background/Objectives: Metastatic uveal melanoma (mUM) carries a poor prognosis and limited systemic treatment options. While immune checkpoint inhibitors have improved outcomes in cutaneous melanoma, their activity in mUM remains modest. Tebentafusp has recently emerged as the first therapy to improve overall survival in HLA-A*02:01–positive patients, but effective options for others remain scarce. This study compared the real-world effectiveness and safety of ipilimumab plus nivolumab versus anti-programmed cell death protein 1 (PD-1) monotherapy. Methods: We conducted a retrospective single-center analysis of patients with mUM treated at the National Institute of Oncology, Budapest. Patients received either dual checkpoint inhibition (ipilimumab plus nivolumab) or anti-PD-1 monotherapy (nivolumab or pembrolizumab). Evaluated outcomes included overall survival (OS), progression-free survival (PFS), objective response rate (ORR), disease control rate (DCR), and immune-related adverse events (irAEs). Survival was analyzed using Kaplan–Meier methods, log-rank tests, and Cox regression. Results: Fifty-five patients were included (33 ipilimumab–nivolumab, 22 anti-PD-1). ORR was 21% versus 5%, and DCR was 42% versus 32%, respectively. Median PFS was 5.8 vs. 3.7 months (*p* = 0.053; HR 0.61, 95% CI 0.34–1.09), and median OS was 12.3 vs. 10.6 months (*p* = 0.214; HR 0.66, 95% CI 0.36–1.22). Grade 3–4 irAEs occurred in 48% of patients receiving ipilimumab–nivolumab compared with 9% on monotherapy. No treatment-related deaths were observed. Conclusions: Anti-PD-1 monotherapy demonstrated limited clinical activity, providing little benefit beyond conventional chemotherapy. Dual checkpoint blockade with ipilimumab and nivolumab achieved higher response and disease control rates, albeit with increased toxicity, suggesting a potential benefit for selected patients. Tebentafusp has emerged as an effective option and a new standard of care for a molecularly defined subgroup of HLA-A*02:01–positive patients. However, for the majority of individuals with metastatic uveal melanoma, effective systemic therapies remain an unmet need.

## 1. Introduction

Uveal melanoma (UM) is the most common primary intraocular malignancy in adults, yet it accounts for only 3–5% of all melanoma cases [1].

It arises from melanocytes located within the uveal tract—the iris, ciliary body, and choroid—with approximately 90% of tumors originating in the choroid. UM mainly affects individuals of European ancestry, typically in the sixth decade of life, and is associated with light eye and skin color [1,2]. Despite effective local treatment of the primary tumor, its biological behavior is characterized by a strong tendency for hematogenous dissemination, as the eye lacks lymphatic drainage [2]. The liver represents the predominant site of metastasis, reflecting specific tumor–host interactions within the hepatic microenvironment that facilitate metastatic colonization. At the molecular level, UM differs fundamentally from cutaneous melanoma, being defined by activating mutations in GNAQ or GNA11 and secondary alterations in genes such as BAP1, SF3B1, or EIF1AX, which are strongly linked to prognosis and metastatic potential [3]. In addition, characteristic chromosomal abnormalities—particularly monosomy 3 and gain of chromosome 8q—serve as major cytogenetic predictors of poor outcome [4]. Despite these biological insights, up to half of patients eventually develop metastatic disease. Once metastasis occurs, the prognosis is poor, with a median overall survival of less than one year, highlighting the urgent need for more effective systemic therapies [5]. Unlike in cutaneous melanoma, where immune checkpoint inhibitors (ICIs) have transformed clinical outcomes, their benefit in UM has remained limited [6,7]. Anti-PD-1 monotherapy with nivolumab or pembrolizumab has shown modest response rates and little survival improvement [7]. This limited efficacy is likely related to the low tumor mutational burden, the immune-privileged nature of the eye, and the immunosuppressive tumor microenvironment characteristic of UM [8,9]. Dual checkpoint inhibition with ipilimumab plus nivolumab has been investigated as a strategy to enhance antitumor immune response [10,11,12]. Emerging evidence from retrospective series and multicenter studies suggests higher response rates with the combination, but at the expense of substantially increased toxicity. Given these uncertainties, real-world data remain critical to clarify the relative effectiveness and safety of different ICI regimens in UM. Here, we report a single-center retrospective analysis comparing clinical outcomes of ipilimumab plus nivolumab versus anti-PD-1 monotherapy in patients with metastatic uveal melanoma treated at the National Institute of Oncology, Budapest.

## 2. Materials and Methods

### 2.1. Patient Selection and Data Collection

We conducted a retrospective single-center study of patients with metastatic uveal melanoma treated with immune checkpoint inhibitors at the National Institute of Oncology, Budapest. The patient inclusion period ranged from July 2015 to May 2025, with the last patient initiating immune checkpoint inhibitor therapy on 8 May 2025. Eligible patients had histologically or clinically confirmed uveal melanoma with metastatic disease verified radiologically, histologically, or both, and received either anti-PD-1 monotherapy (nivolumab or pembrolizumab) or combined CTLA-4 plus PD-1 blockade (ipilimumab plus nivolumab). Although anti-PD-1 agents had been available earlier for cutaneous melanoma, their use in uveal melanoma in Hungary became clinically feasible only from 2015, and combination therapy with ipilimumab plus nivolumab entered routine practice in 2022. Consequently, the treatment cohorts represent different time periods, which may introduce temporal biases, as changes in diagnostic practices, supportive care, and access to subsequent therapies over time could have influenced outcomes. The database cut-off date, defining the end of data collection and follow-up, was 15 August 2025, ensuring a minimum follow-up duration of at least three months for all patients.

### 2.2. Treatment Regimens

In the anti-PD-1 monotherapy cohort, pembrolizumab was the predominant agent, administered in 20 of 22 patients (91%), following the approved schedules (initially 2 mg/kg every 3 weeks, later 200 mg every 3 weeks, and most recently 400 mg every 6 weeks). The remaining two patients received nivolumab monotherapy at 3 mg/kg every 2 weeks; both remained on weight-based dosing throughout. In the combination cohort, induction consisted of ipilimumab 3 mg/kg plus nivolumab 1 mg/kg every 3 weeks for four cycles, followed by nivolumab maintenance 480 mg every 4 weeks; one patient continued pembrolizumab after immune-mediated hepatitis.

### 2.3. Endpoints

The evaluated outcomes included overall survival (OS), progression-free survival (PFS), objective response rate (ORR), disease control rate (DCR), and safety. OS was defined as the time from initiation of immune checkpoint inhibitor therapy to death or last follow-up. PFS was defined as the time from treatment initiation to disease progression or death from any cause. ORR was defined as the proportion of patients achieving complete or partial response, and DCR as the proportion achieving complete response, partial response, or stable disease as best overall response (BOR) [13,14]. Tumor responses were assessed by radiological imaging according to RECIST version 1.1 criteria [15]. Safety was evaluated based on the incidence and severity of treatment-related adverse events, graded according to the Common Terminology Criteria for Adverse Events (CTCAE) versions 4.0 and 5.0, depending on the time of treatment [16,17]. Patients who discontinued treatment before the first radiological assessment were classified as not evaluable for response but remained included in survival analyses.

### 2.4. Statistical Analysis

Baseline demographic and clinical characteristics were summarized with descriptive statistics. Continuous variables were expressed as medians with interquartile ranges (IQRs), while categorical variables were reported as counts and percentages. Efficacy outcomes (objective response rate [ORR], disease control rate [DCR], progression-free survival [PFS], and overall survival [OS]) were analyzed as follows: ORR and DCR were presented as counts and percentages, while PFS and OS were estimated using the Kaplan–Meier method, and survival distributions were compared between treatment arms with the log-rank test. Hazard ratios (HRs) with 95% confidence intervals were calculated from Cox proportional hazards models. Safety outcomes were analyzed by comparing the frequency of grade 3–4 immune-related adverse events (irAEs) between groups using Fisher’s exact test, and by comparing overall CTCAE grade distributions with χ^2^ tests. All *p*-values were two-sided, and values <0.05 were considered statistically significant. Statistical analyses were performed using TIBCO Statistica v12.5 (StatSoft, Tulsa, OK, USA).

### 2.5. Use of Generative AI

During the preparation of this manuscript, the authors used ChatGPT (OpenAI, GPT-5) for language refining and clarity improvements. The authors reviewed and edited all AI-assisted content and take full responsibility for the final text.

## 3. Results

### 3.1. Baseline Characteristics

At the data cut-off (15 August 2025), a total of 55 patients were included: 33 in the ipilimumab–nivolumab group and 22 in the anti-PD-1 monotherapy group. Baseline characteristics are summarized in Table 1. Median age at treatment initiation was 64 years (IQR 58–71) with ipilimumab–nivolumab and 64 years (IQR 56–69) with monotherapy. The proportion of patients aged ≥65 years was 48% versus 36%, respectively. Sex distribution was balanced across groups. ECOG performance status was 0–1 in nearly all cases. Most patients received immune checkpoint inhibitors as first-line systemic therapy. In the ipilimumab–nivolumab group, treatment was administered as first-line in 28 patients (85%), second-line in 1 (3%), and subsequent-line in 4 (12%). The median number of prior systemic therapies was 1 (IQR 0–1, range 0–3). In the anti-PD-1 monotherapy group, 17 patients (77%) received treatment in the first line, 3 (14%) in the second, and 2 (9%) in later lines, with a median of 1 prior therapy (IQR 1–1, range 0–4). Prior to immunotherapy, liver-directed therapy had been performed in 14 of 33 patients (42%) in the ipilimumab–nivolumab cohort and in 11 of 22 patients (50%) in the monotherapy cohort. Regarding primary tumor characteristics, laterality was left/right in 52%/48% in the ipilimumab–nivolumab group and 59%/41% in the monotherapy group. Tumor location in the ipilimumab–nivolumab cohort was choroid in 19/33 (58%), ciliary body in 1/33 (3%), choroid+ciliary body in 4/33 (12%), and choroid+iris in 1/33 (3%), while 8/33 (24%) were missing. In the monotherapy cohort, tumors were located in the choroid in 6/22 (27%), ciliary body in 1/22 (5%), and choroid+ciliary body in 4/22 (18%), with 11/22 (50%) missing; no iris-only cases were recorded. The median largest basal diameter was 12 mm (IQR 11–15, range 4–20) with 36% missing data, and the median tumor thickness was 6 mm (IQR 4–9, range 2–17) with 33% missing in the ipilimumab–nivolumab group. In the monotherapy arm, the median basal diameter was 13 mm (IQR 12–18, range 10–20) with 64% missing, and the median thickness was 9 mm (IQR 6–10, range 5–16) with 55% missing. AJCC T categories were T1 12%, T2 24%, T3 12%, T4 9%, and unknown 42% in the combination cohort; T2 5%, T3 14%, T4 9%, and unknown 73% in the monotherapy cohort. Local treatment of the primary tumor most commonly consisted of enucleation (62% vs. 56%) or brachytherapy (30% vs. 42%), with few sequential procedures (8% vs. 2%). HLA-A*02:01 typing was performed only in the ipilimumab–nivolumab cohort (*n* = 13), among whom 8 (62%) were HLA-negative and 5 (38%) positive; HLA status was unavailable for all patients treated with anti-PD-1 monotherapy. Concerning metastatic distribution, most patients presented with hepatic metastases, with liver-only disease in 15/33 (45%) patients in the combination arm and 8/22 (36%) in the monotherapy arm, and extrahepatic-only disease in 2/33 (6%) and 4/22 (18%), respectively. In the ipilimumab–nivolumab group, metastases were located in the liver (51%), lung (20%), bone (8%), lymph nodes (3%), skin (11%), CNS (2%), and other sites (5%). In the anti-PD-1 monotherapy group, the corresponding frequencies were liver (50%), lung (22%), bone (8%), lymph nodes (8%), skin (0%), CNS (0%), and other (11%). Elevated LDH (>2× ULN) was observed in 8/33 (24%) and 5/22 (23%) patients in the two cohorts, respectively, while markedly elevated LDH (>5× ULN) was observed in 4/33 (12%) patients receiving ipilimumab–nivolumab, while it was absent among those treated with monotherapy.

### 3.2. Treatment Exposure

In the ipilimumab–nivolumab group, 21 of 33 patients (64%) completed all four induction cycles, and 18 (55%) proceeded to maintenance therapy. In most cases, nivolumab was continued; in one patient, pembrolizumab was administered as maintenance after induction. The number of maintenance cycles varied widely. Across the entire cohort (*n* = 33), the median number of cycles was 1 (IQR 0–5; range 0–32), with a mean of 5.2 cycles, reflecting that many patients discontinued early. When restricted to the 18 patients who received at least one maintenance dose, the median number of cycles was 5 (IQR 3–10.5; range 1–32), with a mean of 9.6 cycles, indicating that a subset was able to continue treatment for prolonged periods. Median treatment duration in the combination arm was 4.2 months (IQR 1.4–7.7). Treatment modifications due to toxicity were common, with a substantial proportion requiring interruptions or discontinuation. In the anti-PD-1 group, pembrolizumab was the predominant agent, administered in 20 of 22 patients (91%), with the remainder receiving nivolumab. Treatment schedules followed evolving clinical practice, transitioning from weight-based to fixed-dose regimens over time. Median treatment duration with anti-PD-1 monotherapy was 2.9 months (IQR 2.1–4.7). Treatment interruptions occurred in 2 patients, while most patients continued therapy without major modifications.

### 3.3. Efficacy Outcomes

Objective response rate (ORR) was 7/33 (21%) with ipilimumab–nivolumab compared with 1/22 (5%) with anti-PD-1 monotherapy. Disease control rate (DCR) was 14/33 (42%) versus 7/22 (32%), respectively (Table 2).

In the ipilimumab–nivolumab arm, five patients were not evaluable for radiological response: three died before the first scheduled imaging, one was still on treatment at the data cut-off with the first assessment scheduled beyond that date, and one died from a non-melanoma-related cause. In contrast, all patients in the monotherapy arm had evaluable radiological follow-up.

**Figure 1:**
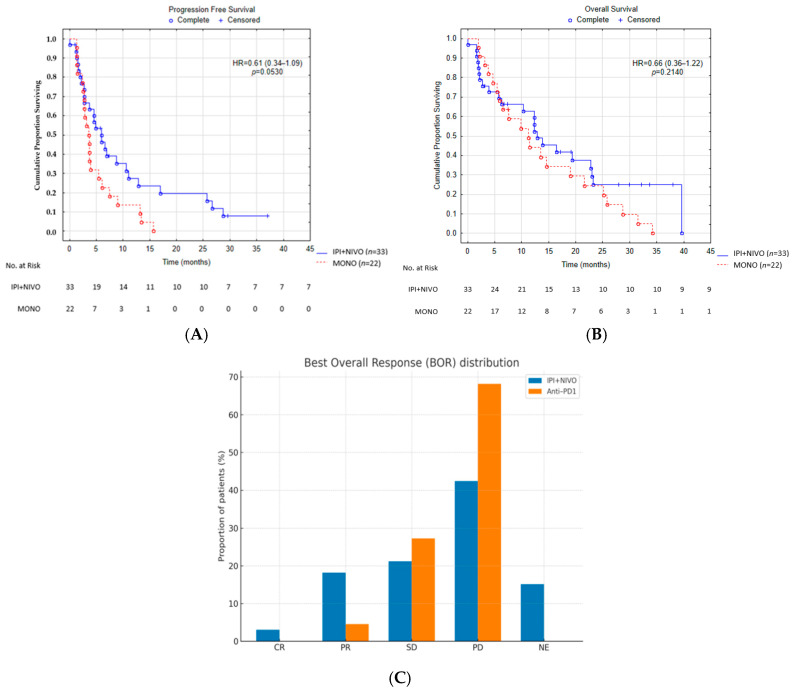
Kaplan–Meier survival and response outcomes in patients treated with ipilimumab plus nivolumab (IPI + NIVO, *n* = 33) versus anti-PD-1 monotherapy (*n* = 22): (**A**) progression-free survival (PFS) with numbers at risk, (**B**) overall survival (OS) with numbers at risk, and (**C**) distribution of best overall response (BOR), including complete response (CR), partial response (PR), stable disease (SD), progressive disease (PD), and not evaluable (NE).

Median progression-free survival (PFS) was 5.8 months (95% CI 3.9–7.9) in the combination arm compared with 3.7 months (95% CI 2.4–5.3) in the monotherapy arm, corresponding to a hazard ratio (HR) of 0.61 (95% CI 0.34–1.09; *p* = 0.053; Figure 1A). Median overall survival (OS) was 12.3 months (95% CI 9.4–16.7) versus 10.6 months (95% CI 7.9–14.8), with an HR of 0.66 (95% CI 0.36–1.22; *p* = 0.214; Figure 1B). The distribution of best overall response (BOR) is illustrated in Figure 1C.

### 3.4. Safety

**Figure 2:**
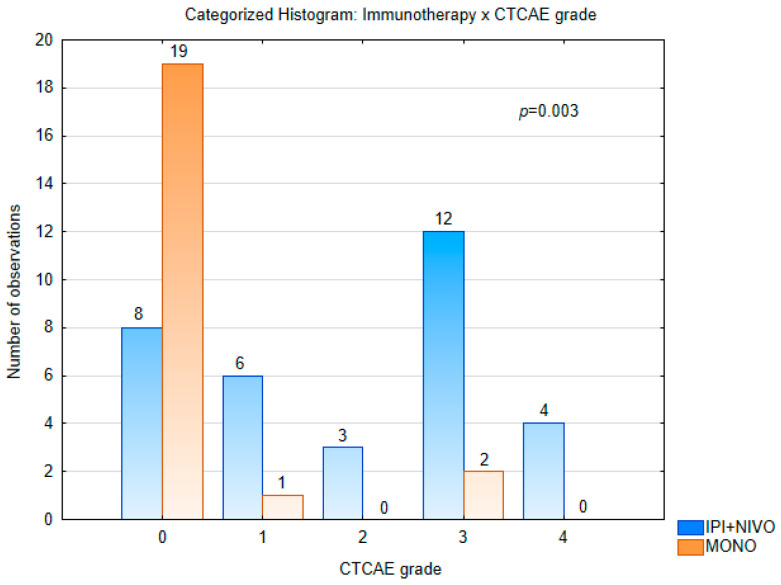
Distribution of treatment-related adverse events by CTCAE grade in patients treated with ipilimumab plus nivolumab (IPI + NIVO, *n* = 33) versus anti–PD-1 monotherapy (*n* = 22). Higher-grade toxicities (grade ≥3) were more frequent with combination therapy (*p* = 0.003).

Immune-related adverse events (irAEs) were more frequent in patients treated with ipilimumab–nivolumab than in those receiving anti-PD-1 monotherapy. In the combination group, 25 of 33 patients (76%) experienced at least one irAE, compared with 3 of 22 patients (14%) in the monotherapy group (*p* < 0.001). Grade 3–4 irAEs occurred in 16/33 (48%) patients in the combination arm compared with 2/22 (9%) in the monotherapy arm (Fisher’s exact *p* = 0.003). The overall distribution of CTCAE grades also differed significantly between treatment arms (χ^2^ *p* = 0.0003; Figure 2). Treatment discontinuation due to toxicity occurred in 8 patients (24%) in the ipilimumab–nivolumab arm and in none of the anti-PD-1 patients. Temporary treatment interruption or modification was required in 6/33 patients (18%) in the combination group and in 2/22 (9%) in the monotherapy group, while the majority continued treatment without impact (58% vs. 91%). No treatment-related deaths were observed. A summary of treatment-related toxicities is shown in Figure 2, with further details by organ system provided in Supplementary Table S1.

### 3.5. Post–Treatment Therapy and Survival Status

**Figure 3:**
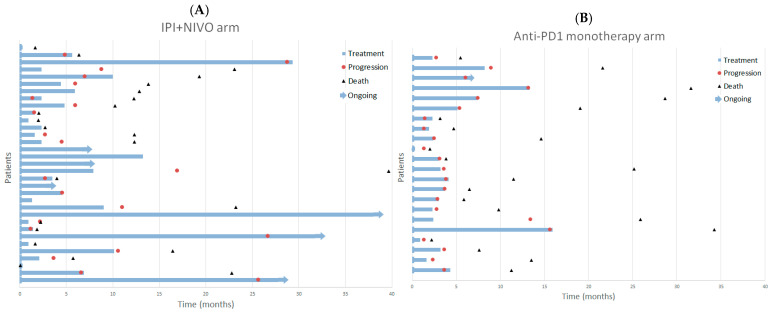
Swimmer plots illustrating treatment duration and key clinical events in patients with metastatic uveal melanoma treated with immune checkpoint inhibitors. Each horizontal bar represents one patient, showing time from treatment initiation to last dose or event. Blue bars indicate treatment duration, arrows denote ongoing therapy, red circles indicate disease progression, and black triangles represent death. (**A**) Ipilimumab plus nivolumab cohort. (**B**) Anti-PD-1 monotherapy cohort.

Following immune checkpoint inhibitor therapy, subsequent chemotherapy was administered more frequently in the anti-PD-1 group than in the ipilimumab–nivolumab group (15/22, 68% vs. 13/33, 39%). At the data cut-off, 10 of 33 patients (30%) in the combination arm and 1 of 22 (4.5%) in the monotherapy arm were alive. These crude survival proportions are not directly comparable due to the non-contemporaneous nature of the cohorts and unequal follow-up duration. Treatment duration and key clinical events are illustrated in Figure 3.

### 3.6. Survival Outcomes

Landmark analyses at 6 and 12 months are summarized in Table 3. The Kaplan–Meier estimated PFS rates with ipilimumab–nivolumab were 49.8% and 27.4%, compared with 22.7% and 13.4% for anti-PD-1 monotherapy. Corresponding OS rates were 66.3% and 63.2% with combination therapy, and 68.2% and 44.1% with monotherapy (Figure 1). At these timepoints, the proportions of patients still receiving treatment were 36.4% and 15.2% in the combination arm, compared with 22.7% and 9.1% in the monotherapy arm.

## 4. Discussion

In our single-center cohort, the clinical activity of anti-PD-1 monotherapy was minimal, consistent with previous evidence showing little meaningful benefit in metastatic uveal melanoma (mUM). Objective responses were rare, disease control was limited, and survival outcomes only marginally exceeded those historically observed with chemotherapy [18,19,20]. In contrast, the addition of CTLA-4 blockade enhanced antitumor activity, with combination therapy inducing objective tumor regression and more durable disease stabilization, in line with results from larger retrospective and phase II studies [6,10,11,21]. Across all efficacy endpoints in our analysis (ORR, DCR, PFS, OS), outcomes were numerically superior with the combination compared with PD-1 inhibition alone. The absence of statistical significance likely reflects the relatively small sample size rather than absence of a true difference. Importantly, across published studies, including both multicenter and single-center analyses, no statistically significant survival advantage has been demonstrated for dual checkpoint blockade compared with PD-1 monotherapy in metastatic UM, although all efficacy endpoints consistently showed numerical benefit in favor of the combination [6,21]. This stands in contrast to cutaneous melanoma, where PD-1 inhibitors alone are highly effective and combined CTLA-4/PD-1 blockade further improves response and survival [22]. Notably, in our cohort, the two patients in the ipilimumab–nivolumab arm who presented with extrahepatic-only metastases were both alive at the time of data cut-off and exhibited prolonged overall survival compared with the median. This observation supports previous evidence indicating that the absence of liver involvement is one of the most favorable prognostic factors in metastatic uveal melanoma [23,24]

The limited evidence base also reflects that patients with UM were systematically excluded from pivotal melanoma trials, leaving retrospective and real-world studies as the main source of clinical data in this setting.

The safety profile observed in our cohort confirms the substantially higher toxicity burden associated with dual checkpoint inhibition compared with PD-1 monotherapy. More than three-quarters of patients receiving ipilimumab plus nivolumab experienced at least one immune-related adverse event (irAE; 76% vs. 14%, *p* < 0.001), and nearly half developed grade 3–4 toxicities (48% vs. 9%, *p* = 0.003). Treatment discontinuation due to toxicity occurred in 24% of patients, a rate somewhat lower than reported in larger multicenter series. In the retrospective analysis by Ciernik et al. [25], including 131 patients from five centers, irAEs occurred in 80.2% of patients treated with ipilimumab plus nivolumab, with grade 3–4 events in 42% and treatment discontinuation due to toxicity in 31.3%. In our cohort, anti-PD-1 monotherapy was associated with a more favorable tolerability profile, with irAEs occurring in only 14% of patients and grade 3–4 events in fewer than 10%, and no treatment discontinuations were observed. Notably, adverse events with monotherapy were almost exclusively endocrine in nature, whereas the combination regimen resulted in a broader spectrum of toxicities, most commonly involving the gastrointestinal, dermatologic, and endocrine systems (Supplementary Table S1). These findings are consistent with data from pivotal trials in cutaneous melanoma, such as CheckMate 067 [22], which reported grade 3–4 irAEs in approximately 55% of patients treated with the combination versus 16% with nivolumab monotherapy. Similarly, retrospective analyses in metastatic uveal melanoma, including the multicenter study by Najjar et al. [21], confirmed higher toxicity rates with dual checkpoint blockade compared with PD-1 inhibition alone. Together, these findings reinforce prior evidence and underscore the need for careful patient selection and close monitoring when using combination immunotherapy in mUM. In both treatment arms, the majority of patients received immune checkpoint inhibitors as first-line systemic therapy (85% in the ipilimumab–nivolumab group and 77% in the anti-PD-1 group). This indicates that most patients were treatment-naïve at the start of immunotherapy, limiting the potential confounding effect of prior systemic therapies.

Our findings also have important implications for clinical practice in mUM, a disease with limited therapeutic options and poor overall prognosis. While dual checkpoint inhibition with ipilimumab plus nivolumab achieved higher response and disease control rates than PD-1 monotherapy, the survival advantage remained modest and was accompanied by substantially greater toxicity. For patients who are not eligible for HLA-restricted therapy, combined checkpoint blockade currently represents the most active immunotherapeutic option, albeit with significant risks that require careful patient selection. Tebentafusp, by contrast, has established itself as the first systemic therapy to demonstrate a consistent and durable overall survival benefit in HLA-A*02:01-positive patients, as confirmed in the phase III IMCgp100–202 trial and its three-year follow-up [26,27]. While dual checkpoint inhibition may provide incremental improvements over PD-1 monotherapy, the true therapeutic breakthrough in mUM to date has been achieved with tebentafusp [21,26,27].

This study has several limitations. First, it was retrospective and conducted at a single institution, which carries the possibility of selection and interpretation biases and limits the generalizability of the findings. Second, the number of patients was relatively small, reducing statistical power and preventing more detailed subgroup analyses. Third, the treatment groups were not contemporaneous: most patients in the combination arm were treated after 2021, while monotherapy was mainly used earlier. This difference may have introduced distortions related to changes in supportive care or the availability of later treatment options. Finally, follow-up duration was not equal between the two cohorts, which could affect survival estimates. To reduce these limitations, we reported reverse Kaplan–Meier follow-up times and included 6- and 12-month survival rates in addition to medians. Despite these limitations, our results add important real-world data in mUM, where prospective evidence remains limited.

Although dual checkpoint inhibition has modestly improved outcomes compared with monotherapy, most patients still experience limited long-term benefit, although tebentafusp has emerged as an effective option for HLA-A*02:01–positive disease. Given the predominant hepatic involvement in this malignancy, liver-directed therapies (LDTs) remain an important component of multimodal management. Systematic reviews have shown that approaches such as transarterial chemoembolization, selective internal radiation therapy, and isolated hepatic perfusion can provide meaningful disease control in selected patients [28]. In our institutional experience, intra-arterial chemotherapy in 50 patients with hepatic metastases achieved a median PFS of 7 months and an estimated OS of 11 months [29]. These retrospective data provide useful historical context but should be interpreted cautiously given the non-prospective design. Of note, the CHOPIN trial has provided the first prospective evidence supporting this combined approach. In its phase Ib part, published by Tong et al. [30], the combination of melphalan percutaneous hepatic perfusion with ipilimumab plus nivolumab demonstrated encouraging safety and early efficacy in advanced uveal melanoma. Updated phase II results presented by Kapiteijn et al. at the European Society for Medical Oncology (ESMO) Congress 2025, Berlin, Germany [31], showed significant improvements in one-year progression-free survival (54.7% vs. 15.8%; *p* < 0.001), overall survival (23.1 vs. 19.6 months; *p* = 0.006), and best overall response rate (76.3% vs. 39.5%; *p* < 0.001) compared with hepatic perfusion alone, with manageable toxicity. These data reinforce the rationale for integrating liver-directed and systemic immunotherapeutic strategies in metastatic uveal melanoma, including emerging agents such as tebentafusp.

Our cohort of 55 patients represents one of the largest single-center, real-world series from a national referral institution with nationwide coverage. Despite the inherent limitations of retrospective design and modest sample size, our findings are consistent with recently published international data, including the meta-analysis by Yamada et al. [32], which confirmed the superior efficacy of dual checkpoint blockade over PD-1 monotherapy in metastatic uveal melanoma.

A major advance in recent years has been the development of tebentafusp, the first systemic therapy to significantly improve overall survival in HLA-A*02:01–positive patients, as shown in the pivotal IMCgp100–202 trial and its long-term follow-up [22,23]. However, its benefit remains restricted to a molecularly defined subgroup, and access is limited in many countries. Future studies should clarify how tebentafusp can be best combined or sequenced with checkpoint inhibitors and liver-directed therapies. In parallel, biomarker-driven approaches such as HLA typing and molecular profiling may help tailor treatment more precisely. Given the lack of dedicated prospective trials in UM, additional real-world and prospective investigations remain essential to optimize therapy.

## 5. Conclusions

In this single-center retrospective study of metastatic uveal melanoma, dual checkpoint blockade with ipilimumab plus nivolumab achieved higher response and disease control rates than PD-1 inhibitor monotherapy, albeit with substantially greater toxicity. As PD-1 monotherapy shows only limited activity and offers outcomes comparable to those historically seen with chemotherapy, it cannot be regarded as a viable standalone option. While tebentafusp represents a therapeutic advance for HLA-A*02:01–positive patients, effective systemic treatments remain limited for the broader population. Liver-directed approaches are likely to continue serving as valuable components of multimodal management, complementing emerging systemic strategies aimed at improving outcomes in this challenging disease.

## Figures and Tables

**Table 1:** Baseline characteristics of patients treated with IPI + NIVO versus anti-PD-1 monotherapy.

Characteristic	IPI + NIVO (*n* = 33)	Anti-PD-1 (*n* = 22)
Median age, years (IQR)	64 (58–71)	64 (56–69)
Age <65/≥65	17 (52%)/16 (48%)	14 (64%)/8 (36%)
Sex (male/female)	16 (48%)/17 (52%)	11 (50%)/11 (50%)
ECOG performance status 0–1/≥2	32 (97%)/1 (3%)	22 (100%)/0 (0%)
Line of immunotherapy		
First line	28 (85%)	17 (77%)
Second line	1 (3%)	3 (14%)
Later lines	4 (12%)	2 (9%)
Median no. of prior systemic therapies (IQR, range)	1 (0–1, 0–3)	1 (1–1, 0–4)
Prior liver-directed therapy	14 (42%)	11 (50%)
Primary tumor laterality (left/right)	52%/48%	59%/41%
Primary tumor location		
Choroid	19 (58%)	6 (27%)
Ciliary body	1 (3%)	1 (5%)
Choroid + ciliary body	4 (12%)	4 (18%)
Choroid + iris	1 (3%)	0 (0%)
Unknown/missing	8 (24%)	11 (50%)
Median largest basal diameter, mm (IQR, range)	12 (11–15, 4–20); 36% missing	13 (12–18, 10–20); 64% missing
Median tumor thickness, mm (IQR, range)	6 (4–9, 2–17); 33% missing	9 (6–10, 5–16); 55% missing
AJCC T category	T1 12%, T2 24%, T3 12%, T4 9%, Unknown 42%	T2 5%, T3 14%, T4 9%, Unknown 73%
Local treatment of primary tumor		
Enucleation	62%	56%
Brachytherapy	30%	42%
Sequential/other	8%	2%
HLA-A*02:01 status	Negative 8 (62%), Positive 5 (38%), Unknown 20 (61%)	Unknown (100%)
Metastatic pattern		
Liver-only disease	15 (45%)	8 (36%)
Extrahepatic-only disease	2 (6%)	4 (18%)
Sites of metastases		
Liver	51%	50%
Lung	20%	22%
Bone	8%	8%
Lymph nodes	3%	8%
Skin	11%	0%
CNS	2%	0%
Other	5%	11%
LDH ≤ 2× ULN/>2× ULN/>5× ULN	25 (76%)/8 (24%)/4 (12%)	17 (77%)/5 (23%)/0 (0%)

Data are presented as median (interquartile range, IQR) for continuous variables and as number (percentage) for categorical variables. ULN, upper limit of normal; LDH, lactate dehydrogenase; ECOG, Eastern Cooperative Oncology Group.

**Table 2:** Best overall response (BOR) in patients treated with ipilimumab plus nivolumab (IPI+NIVO) or anti-PD-1 monotherapy, according to RECIST v1.1 criteria.

Outcome	IPI + NIVO (*n* = 33)	Anti-PD-1 (*n* = 22)
CR	1 (3%)	0 (0%)
PR	6 (18%)	1 (5%)
SD	7 (21%)	6 (27%)
PD	14 (42%)	15 (68%)
NE	5 (15%)	0 (0%)
ORR (CR + PR)	7 (21%)	1 (5%)
DCR (CR + PR + SD)	14 (42%)	7 (32%)
Median PFS, months	5.8	3.7
Median OS, months	12.3	10.6
HR (95% CI), *p*—PFS	0.61 (0.34–1.09), *p* = 0.053	
HR (95% CI), *p*—OS	0.66 (0.36–1.22), *p* = 0.214	

ORR, objective response rate; DCR, disease control rate; CR, complete response; PR, partial response; SD, stable disease; PD, progressive disease; NE, not evaluable; PFS, progression-free survival; OS, overall survival; HR, hazard ratio; CI, confidence interval.

**Table 3:** Landmark survival rates at 6 and 12 months in patients treated with IPI+NIVO versus anti-PD-1 monotherapy.

Cohort	6-mo PFS Rate	12-mo PFS Rate	6-mo OS Rate	12-mo OS Rate	On Treatment ≥ 6 mo	On Treatment ≥ 12 mo
IPI + NIVO (*n* = 33)	49.8%	27.4%	66.3%	63.2%	36.4%	15.2%
Anti–PD–1 (*n* = 22)	22.7%	13.4%	68.2%	44.1%	22.7%	9.1%

Rates derived from Kaplan–Meier estimates; treatment exposure based on observed duration data. PFS = progression-free survival; OS = overall survival.

## Data Availability

The data presented in this study are available upon reasonable request from the corresponding author.

## Supplementary Materials

The following supporting information can be downloaded at https://www.mdpi.com/article/10.3390/cancers17213521/s1. Table S1: Detailed summary of immune-related adverse events by organ system in patients treated with ipilimumab plus nivolumab versus anti-PD-1 monotherapy.

## Abbreviations

The following abbreviations are used in this manuscript:

UM	Uveal melanoma
mUM	Metastatic uveal melanoma
ICI	Immune checkpoint inhibitor
irAE	Immune-related adverse event
OS	Overall survival
PFS	Progression-free survival
ORR	Objective response rate
DCR	Disease control rate
CR	Complete response
PR	Partial response
SD	Stable disease
PD	Progressive disease
NE	Not evaluable
CI	Confidence interval
HR	Hazard ratio
LDH	Lactate dehydrogenase
ECOG	Eastern Cooperative Oncology Group
CTCAE	Common Terminology Criteria for Adverse Events
ULN	Upper limit of normal
BOR	Best overall response
KM	Kaplan–Meier
