# Supplementary material for: Real-World Outcomes of Ipilimumab–Nivolumab vs. Anti-PD-1 Monotherapy in Metastatic Uveal Melanoma: A Single-Center Retrospective Study
Source: Cancers (Basel). 2025 Oct 31;17(21):3521. doi: 10.3390/cancers17213521 (PMC12609781; doi:10.3390/cancers17213521)
Supplement: Supplementary file 1 [file cancers-17-03521-s001.zip › cancers-3924299-supplementary.pdf]

Supplementary Materials: Real-World Outcomes of Ipilimumab–Nivolumab vs. Anti-PD-1 Monotherapy in Metastatic Uveal Melanoma: A Single-Center Retrospective Study

Gitta Pánczél, Patrik Horváth, Erijona Temaj, Kata Czirbesz, Mihály Tamás Kispál, Georgina Fröhlich and Tímea Balatoni

Table S1. Detailed summary of immune-related adverse events by organ system in patients treated with ipilimumab plus nivolumab versus anti-PD-1 monotherapy.

Organ system	IPI+NIVO (*n* = 33)	Anti-PD-1 (*n* = 22)
Dermatologic	4 (12%) / 0 Gr3–4	0 (0%) / 0 Gr3–4
Dermatologic, Endocrine	1 (3%) / 1 Gr3–4	0 (0%) / 0 Gr3–4
Endocrine	3 (9%) / 1 Gr3–4	2 (9%) / 1 Gr3–4
Endocrine, Gastrointestinal	2 (6%) / 2 Gr3–4	0 (0%) / 0 Gr3–4
Endocrine, Hepatobiliary	1 (3%) / 1 Gr3–4	0 (0%) / 0 Gr3–4
Endocrine, Metabolism and nutrition	0 (0%) / 0 Gr3–4	1 (5%) / 1 Gr3–4
Gastrointestinal	5 (15%) / 4 Gr3–4	0 (0%) / 0 Gr3–4
Gastrointestinal, Respiratory	1 (3%) / 1 Gr3–4	0 (0%) / 0 Gr3–4
Hepatobiliary, Dermatologic	1 (3%) / 1 Gr3–4	0 (0%) / 0 Gr3–4
Hepatobiliary, Endocrine, Dermatologic	1 (3%) / 1 Gr3–4	0 (0%) / 0 Gr3–4
Hepatobiliary, Gastrointestinal	1 (3%) / 1 Gr3–4	0 (0%) / 0 Gr3–4
Musculoskeletal	1 (3%) / 1 Gr3–4	0 (0%) / 0 Gr3–4
Musculoskeletal, Dermatologic	1 (3%) / 0 Gr3–4	0 (0%) / 0 Gr3–4
Musculoskeletal, General, Dermatologic, Hepatobiliary	1 (3%) / 1 Gr3–4	0 (0%) / 0 Gr3–4
Renal, Endocrine	1 (3%) / 1 Gr3–4	0 (0%) / 0 Gr3–4
Respiratory	1 (3%) / 0 Gr3–4	0 (0%) / 0 Gr3–4
None	8 (24%) / 0 Gr3–4	19 (86%) / 0 Gr3–4

Values are shown as numbers (percentage of total patients); Grade 3–4 events are indicated separately within each organ system. irAE = immune-related adverse event.
